# Supplementary material for: Serum expression of Vascular Endothelial-Cadherin, CD44, Human High mobility group B1, Kallikrein 6 proteins in different stages of laryngeal intraepithelial lesions and early glottis cancer
Source: PeerJ. 2022 Apr 19;10:e13104. doi: 10.7717/peerj.13104 (PMC9029362; doi:10.7717/peerj.13104)
Supplement: Supplemental Information 2 — * - Mann-Whitney U test # - Kruskal Wallis one-way analysis of variance [file peerj-10-13104-s002.docx]

| **Variable** | **HMGB1** | | | | |
| --- | --- | --- | --- | --- | --- |
|  | **Mean ± SD** | **p-value** | **Test statistic** | **Degrees of freedom** | **Effect size** |
| **Gender**  **Female**  **Male** | 69.55 ± 67.8  44.24 ± 47.12 | 0.243* | 575 | 1 | 0.0171 |
| **Histopathology**  **Invasive cancer**  **High grade dysplasia**  **Low grade dysplasia**  **No dysplasia** | 41.52 ± 40.98  74.27 ± 80.29  81.14 ± 92.64  54.5 ± 54.03 | 0.897^#^ | 0.597 | 3 | 0.0076 |
| **Smoking**  **Non-smokers**  **Smokers** | 49.31 ± 53.7  54.1 ± 56.92 | 0.645* | 657.5 | 1 | 0.0026 |
| **GERD**  **No**  **Yes** | 50.27 ± 54.82  62.27 ± 59.82 | 0.23* | 361.5 | 1 | 0.018 |
| **Variable** | **KLK6** | | | | |
|  | **Mean ± SD** | **p-value** | **Test statistic** | **Degrees of freedom** | **Effect size** |
| **Gender**  **Female**  **Male** | 17.61 ± 27.06  8.59 ± 19.81 | 0.493* | 621.5 | 1 | 0.0059 |
| **Histopathology**  **Invasive cancer**  **High grade dysplasia**  **Low grade dysplasia**  **No dysplasia** | 7.28 ± 18.49  20.43 ± 31  24.33 ± 35.39  11.82 ± 21.34 | 0.354^#^ | 3.258 | 3 | 0.0412 |
| **Smoking**  **Non-smokers**  **Smokers** | 9.84 ± 20.24  12.42 ± 23.96 | 0.405* | 621.5 | 1 | 0.0087 |
| **GERD**  **No**  **Yes** | 10.45 ± 22.1  16.61 ± 25.33 | 0.06* | 308.5 | 1 | 0.0443 |
| **Variable** | **VE-cadherin** | | | | |
|  | **Mean ± SD** | **p-value** | **Test statistic** | **Degrees of freedom** | **Effect size** |
| **Gender**  **Female**  **Male** | 9.35 ± 15.24  5.27 ± 11.19 | 1* | 687.5 | 1 | 0 |
| **Histopathology**  **Invasive cancer**  **High grade dysplasia**  **Low grade dysplasia**  **No dysplasia** | 4.58 ± 10.47  11.95 ± 17.42  14.17 ± 20.01  6.1 ± 11.76 | 0.1^#^ | 6.261 | 3 | 0.0792 |
| **Smoking**  **Non-smokers**  **Smokers** | 5.97 ± 11.59  6.96 ± 13.35 | 0.22* | 583.5 | 1 | 0.0188 |
| **GERD**  **No**  **Yes** | 6.51 ± 12.78  7.14 ± 12.8 | 0.418* | 392 | 1 | 0.0082 |
| **Variable** | **CD44** | | | | |
|  | **Mean ± SD** | **p-value** | **Test statistic** | **Degrees of freedom** | **Effect size** |
| **Gender**  **Female**  **Male** | 2.11 ± 1.4  2.13 ± 1.19 | 0.51* | 624 | 1 | 0.0054 |
| **Histopathology**  **Invasive cancer**  **High grade dysplasia**  **Low grade dysplasia**  **No dysplasia** | 1.92 ± 1.08  1.77 ± 0.72  2.29 ± 0.94  2.49 ± 1.58 | 0.433^#^ | 2.741 | 3 | 0.0347 |
| **Smoking**  **Non-smokers**  **Smokers** | 2.3 ± 1.49  2.04 ± 1.13 | 0.706* | 665.5 | 1 | 0.0018 |
| **GERD**  **No**  **Yes** | 2.22 ± 1.29  1.72 ± 1.05 | 0.084* | 320.5 | 1 | 0.0373 |
